# The therapeutic potential of exercise for improving mobility in multiple sclerosis

**DOI:** 10.3389/fphys.2024.1477431

**Published:** 2024-09-13

**Authors:** Giuseppe Locatelli, Martin Stangel, Daniel Rooks, Julian Boesch, Eliane Pierrel, Serge Summermatter

**Affiliations:** ^1^ Immunology Disease Area, Biomedical Research, Novartis Pharma AG, Basel, Switzerland; ^2^ Translational Medicine, Biomedical Research, Novartis Pharma AG, Basel, Switzerland; ^3^ Translational Medicine, Biomedical Research, Novartis Pharma AG, Cambridge, MA, United States; ^4^ Diseases of Aging and Regenerative Medicine, Biomedical Research, Novartis Pharma AG, Basel, Switzerland

**Keywords:** muscle weakness, motor fatigability, multiple sclerosis, resistance exercise, aerobic exercise

## Abstract

Multiple sclerosis (MS) is a chronic autoimmune disease characterized by inflammation and demyelination in the central nervous system (CNS) with subsequent axonal and neuronal degeneration. These changes are associated with a broad range of symptoms including skeletal muscle dysfunction. Importantly, musculoskeletal impairments manifest in various ways, compromise the quality of life and often precede the later development of mobility disability. As current standard disease modifying therapies for MS predominantly act on neuroinflammation, practitioners and patients face an unmet medical need for adjunct therapies specifically targeting skeletal muscle function. This review is intended to detail the nature of the skeletal muscle dysfunctions common in people with MS (pwMS), describe underlying intramuscular alterations and outline evidence-based therapeutic approaches. Particularly, we discuss the emerging role of aerobic and resistance exercise for reducing the perception of fatigue and increasing muscle strength in pwMS. By integrating the most recent literature, we conclude that both exercise interventions should ideally be implemented as early as possible as they can address MS-specific muscle impairments. Aerobic exercise is particularly beneficial for pwMS suffering from fatigue and metabolic impairments, while resistance training efficiently counters muscle weakness and improves the perception of fatigue. Thus, these lifestyle interventions or possible pharmacological mimetics have the potential for improving the general well-being and delaying the functional declines that are relevant to mobility.

## Introduction

MS is a chronic inflammatory disease characterized by immune cell infiltration into the CNS, diffuse glial activation, demyelination and progressive neurodegeneration in the brain and spinal cord (Disanto et al., 2016; McGinley et al., 2021; Thomas et al., 2022). The first symptoms commonly present during young adulthood, with patients usually classified into four main subtypes: Clinically Isolated Syndrome (CIS), Relapsing-Remitting Multiple Sclerosis (RRMS), Secondary Progressive Multiple Sclerosis (SPMS) and Primary Progressive Multiple Sclerosis (PPMS) (Disanto et al., 2016; McGinley et al., 2021; Barile et al., 2022). CIS describes an initial neurologic symptom lasting at least 24 h with a high risk of evolving into RRMS, characterized by repeated episodes of symptom exacerbations followed by remission. RRMS accounts for approximately 85% of all MS cases (Ruiz et al., 2019; van der Feen et al., 2020). Following a relapse, the symptoms may again disappear without causing any additional disability, or only partially remit resulting in heterogeneous patterns of disease progression. In approximately 50% of RRMS patients, the disease will turn into a progressive form over time, and is referred to as SPMS (McGinley et al., 2021). The remaining 15% of patients display a slowly progressive disease without phases of recovery (Ruiz et al., 2019). They are therefore diagnosed with PPMS. However, while these subtype definitions are still used in clinical trials and medical practice, a new scientific consensus has emerged indicating MS as a continuously progressive disease along the clinical spectrum (Vollmer et al., 2021).

Despite such heterogeneous disease progression, the various forms share important commonalities. In fact, more than 90% of pwMS are affected by mobility impairments within the first 10 years post-diagnosis and these impairments constitute the major cause of disability (Hemmett et al., 2004; Baird et al., 2018; van der Feen et al., 2020). The extent of motor impairment is often quantified by the Expanded Disability Status Scale (EDSS) instrument with scores ranging from 0 (normal) to 10 (death due to MS). Patients with an EDSS of two display mild disability; those with an EDSS above 4, severe ambulatory deficits; from 6 on, walking needs to be aided by assistive devices (Buzzard et al., 2012). People affected by MS perceive falls and difficulties getting out into the community as their most debilitating limitations (Plummer et al., 2023). Besides fatigue, such mobility constraints constitute a main reason for job loss (Julian et al., 2008).

Exercise is a well-known intervention for diseases that affect the skeletal muscle (Cento et al., 2022). However, most of the earlier research explicitly investigating the effects of exercise on skeletal muscle in pwMS suffers from considerable limitations. For instance, many exercise studies in pwMS are underpowered due to relatively small trials with few patients. On top of that, most studies did not scrutinize the histological and molecular skeletal muscle changes, their potential link to mobility and their responsiveness to exercise interventions. Hence, a better understanding of the musculoskeletal changes that underlie the mobility disability in MS is one crucial first step towards developing novel therapeutic approaches to alleviate disease burden.

## Muscle dysfunction in MS is characterized by muscle weakness and increased fatigability

Axon degeneration is the primary cause of musculoskeletal symptoms in MS. As a result of the demyelinating process in the CNS, the neuromuscular recruitment and motor unit firing rates decrease (Dorfman et al., 1989; Rice et al., 1992). Eventually, the insufficient neural input attenuates force production in skeletal muscle, which manifests as muscle weakness, a key component of muscle dysfunction in MS. A comprehensive meta-analysis of studies assessing muscle strength of upper and lower limb muscles quantified the reductions in strength as 10%–60% (Jørgensen et al., 2017). Healthy, untrained adults normally generate average maximal forces of 2.38 (females) and 3.19 Nm/kg (males) during knee extension (Šarabon et al., 2021); a movement that is carried out predominantly by the quadriceps muscle and that is essential to execute activities of daily living such as walking. A quadriceps strength that falls below 3 Nm/kg already represents a risk for impaired function in rising from a chair, usual gait speed, as well as stair ascent and descent (Ploutz-Snyder et al., 2002). Although these latter analyses have been conducted in a different patient population, i.e., elderly, it suggests that the declines in muscle strength frequently reported in pwMS are sufficient to cause a loss of functional independence and autonomy. Besides muscle weakness, which is determined by assessing maximal strength, skeletal muscles of pwMS have a decreased ability to maintain force-generating contractions over an extended period. This phenomenon, referred to as increased motor fatigability or reduced motor endurance, is defined as a reduced capacity to produce and maintain voluntary or evoked force during physical activity (Royer et al., 2022). Increased motor fatigability does not seem to solely result from muscle weakness as suggested by a lack of correlation between the degree of baseline maximal voluntary force output and fatigability (Schwid et al., 1999). Therefore, muscle weakness and increased motor fatigability should be viewed as distinct impairments and suggest the involvement of both type I (endurance) and II (strength) muscle fibers.

In the context of this review, it is furthermore pivotal to differentiate motor fatigability from general fatigue. General fatigue is defined as a “subjective sensation of weariness, an increasing sense of effort, a mismatch between effort expended and actual performance, or exhaustion” (Kluger et al., 2013). Fatigue is one of the most common symptoms of MS and reported by 37%–78% of pwMS across all clinical phenotypes (Oliva Ramirez et al., 2021). Primary fatigue results from direct CNS damage (i.e., lesions of the cortical neurons or the subcortical ascending arousal system); while secondary fatigue is driven by factors that are only indirectly related to MS (e.g., sleep disturbances, chronic urinary tract infections, or side effects of pharmacological interventions) (Patejdl and Zettl, 2022).

Intriguingly, however, neither fatigue nor other impairments in neuromotor transmission can fully explain the reductions in force generation (i.e., muscle weakness and increased motor fatigability) in pwMS. Notably, when evoking contraction directly on muscle by electrical stimulation, thus bypassing the CNS, the decrement in isometric force remains more pronounced in pwMS than in healthy controls (de Haan et al., 2000). These data suggest that muscle-intrinsic factors must additionally contribute to muscle dysfunction in MS. Therefore, the following section of this review focuses on the intramuscular changes that may contribute to muscle weakness and motor fatigability, key hallmarks of MS chiefly linked to the quality of life of these patients (Barin et al., 2018).

## MS affects skeletal muscle fiber size, contractile properties, and metabolic capacity

**FIGURE 1:**
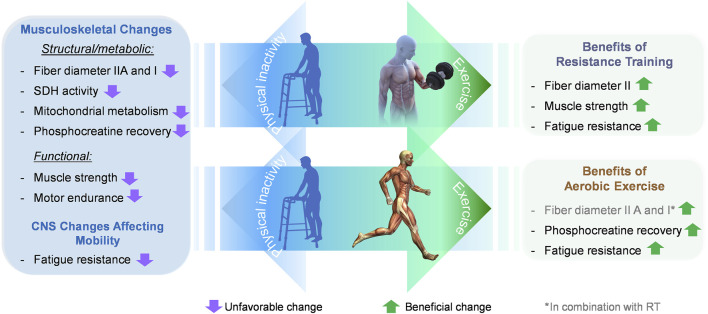
Effect of different exercise modalities on musculoskeletal and CNS changes affecting mobility in pwMS. MS is associated with defined musculoskeletal changes in skeletal muscle. Some of these changes are of structural (atrophy of type I and IIA fibers), while others are of metabolic nature (reduced mitochondrial activity and slowed phosphocreatine recovery). Ultimately, both impairments contribute to muscle dysfunction, i.e., reduced muscle strength and increased motor fatigability (decreased motor endurance). Moreover, changes in the CNS evoke a perception of general fatigue, which leads to reduced physical activity. Resistance training in pwMS promotes hypertrophy of type IIA fibers, increases muscle strength and counters fatigue. Thereby resistance training mends two prominent impairments that reduce mobility, accelerate disability, and decrease the quality of life. Aerobic exercise can restore phosphocreatine recovery and change the patient’s perception of fatigue. The direct comparison of the two exercise modalities highlights that the benefits of resistance exercise in terms of functional improvements are collectively more favorable than those of aerobic exercise.

Neuromuscular diseases affect the peripheral nervous system, neuromuscular junction or skeletal muscle and generally provoke substantial loss of muscle mass and body weight. This does not hold true for MS, which is a disease of the CNS and thus primarily affects the first motor neuron. In fact, body weight is often unaltered in subjects with MS, while they show higher fat and lower lean mass (Hansen et al., 2015; Wingo et al., 2018). More detailed investigations into the lean mass changes point towards lower mass of individual skeletal muscles, such as the rectus femoris and the gastrocnemius muscle (Kirmaci et al., 2022). Further studies revealed that the observed atrophy was due primarily to changes in type I and IIA fibers, although the results for type I fibers are slightly less consistent across studies (Figure 1) (Wens et al., 2014; Hansen et al., 2015). Type I fibers, also referred to as slow-twitch fibers, have a high oxidative but low glycolytic capacity. As a result, they are resistant to localized fatigue, support repetitive mechanical tasks for a prolonged duration, and contribute less to maximal muscle strength (Bottinelli and Reggiani, 2000; van der Zwaard et al., 2021; Serrano et al., 2023). A loss of type I fibers will reduce the ability to sustain physical activity for extended periods of time. On the other end of the fatigue resistance – strength continuum, type IIB fibers have a high glycolytic and low oxidative capacity designed to contribute mostly to muscle force production (strength) and are more easily fatigued. In between are type IIA fibers that contribute to both fatigue resistance and muscle strength and are recruited for tasks requiring greater fatigue resistance and muscle force (Hodson-Tole and Wakeling, 2009). Atrophy of types I and IIA specific fibers are linked to reduced resistance to fatigue and reduced maximal muscle strength, respectively. Strikingly, even when normalizing force measurements to muscle mass and cross-sectional area, the muscle dysfunction associated with MS persists (Lambert et al., 2001; Wens et al., 2014). These data suggest that the atrophy and the loss of specific contractile properties drive the muscle dysfunctions observed in pwMS.

Further insights into the mechanisms that might underlie the musculoskeletal phenotype of MS derive from the metabolic characterization of muscle tissue (Figure 1). Measurements of 31-phosphorus magnetic resonance spectroscopy (^31^P MRS) to evaluate the *in-vivo* turnover of high energy phosphates demonstrate that MS negatively affects muscle metabolism (Kent-Braun et al., 1994). Specifically, phosphocreatine resynthesis is significantly lower in the skeletal muscles of pwMS compared to healthy controls (Kent-Braun et al., 1994; Naëgel et al., 2023). Phosphocreatine serves as a high-energy phosphate donor used to instantly replenish ATP levels anaerobically. Since the muscle stores of ATP are small, this mechanism is crucial to ensure the high rates of ATP resynthesis required for maintaining muscle contraction (Hargreaves and Spriet, 2020). A reduced capacity to restore ATP levels from phosphocreatine forces skeletal muscle to fall back on alternative energy strategies such as oxidative phosphorylation (OXPHOS). This process is governed by five complexes located within the inner mitochondrial membrane. Experiments investigating OXPHOS events in MS reported 22%–40% reductions in succinate dehydrogenase activity (complex II) (Kent-Braun et al., 1985; Spaas et al., 2022) along with diminished protein levels of mitochondrial complex II (Spaas et al., 2022) and I (Kumleh et al., 2006; Spaas et al., 2022). Notably, mitochondrial dysfunction has also been investigated in CNS cells and might be an underlying factor in the progressive neurodegeneration observed in pwMS (Witte et al., 2014). Moreover, studies using near-infrared spectroscopy evaluated the total *in-vivo* mitochondrial capacity of gastrocnemius muscles of pwMS. The metabolic deficits found are in line with altered OXPHOS activity. More precisely, these investigations revealed that overall muscle-specific oxidative metabolism was reduced by ∼33% in pwMS compared to healthy controls (Harp et al., 2016). Taken together, these data underscore a reduced capacity to provide ATP for muscle contraction in pwMS, which helps explain the increased fatigability and muscle weakness.

Since skeletal muscle accounts for up to 40% of total body mass and energy expenditure by muscle is a main contributor to basal systemic metabolic rate, the altered metabolic profile has a direct impact on whole body energy expenditure. In line with diminished mitochondrial capacity, lower oxygen consumption, reflecting lower energy expenditure, has been reported in pwMS compared to healthy controls (Hansen et al., 2015; Spaas et al., 2022). This fact might partially explain the higher propensity for obesity and body fat accretion in pwMS.

Collectively, the current body of evidence suggests that muscle dysfunction in people with MS is not exclusively driven by reduced neural input, but also by altered intramuscular properties.

## Pharmacological interventions to treat MS

The current standard of care for MS consists of a multidisciplinary approach where patients receive disease-modifying therapies (DMTs), symptomatic treatments, psychological support, and rehabilitation. Available DMTs encompass various mechanisms of action and routes of administration. Drugs for RRMS and active SPMS are anti-inflammatory treatments including interferons, glatiramer acetate, teriflunomide, sphingosine 1-phosphate receptor modulators, fumarates, cladribine, and different monoclonal antibodies targeting either CD20 on B cells, leukocyte integrins, or CD52 on leukocytes (McGinley et al., 2021). The only approved DMT for PPMS is ocrelizumab, an anti-CD20 mAb (McGinley et al., 2021; Portaccio et al., 2022). These DMTs reduce clinical relapses and MRI lesions (new T2 lesions, gadolinium-enhancing lesions) and their treatment efficacy, which ranges from 29% to 68%, is evaluated by reductions in annualized relapse rates (McGinley et al., 2021). It is unclear to what extent current DMTs tackle unmet needs of disease progression in the absence of relapses. For instance, interferon treatments reduced the risk of increasing EDSS scores in RRMS but did not significantly slow progression in SPMS (La Mantia et al., 2013).

In contrast to DMT, Fampridine, a potassium-channel blocker aiming at treating walking disabilities in pwMS, improved gait balance in subjects with MS (Hupperts et al., 2022; Ghorbanpour et al., 2023). The drug acts on the central and peripheral nervous systems and is the only pharmacological therapy that is approved for gait imbalance in these patients (Ghorbanpour et al., 2023). A recent meta-analysis including 17 mainly non-placebo-controlled studies, concludes that Fampridine corrects gait imbalances effectively (Ghorbanpour et al., 2023). Given the limited treatment options for musculoskeletal dysfunctions in MS, the medical need for adjunct therapies to restore functional independence persists.

## Non-pharmacological interventions to restore muscle function and mobility

Intriguingly, exercise is a highly efficacious, non-pharmacological intervention to treat musculoskeletal conditions and improve functional parameters (e.g., strength, localized muscle endurance, cardiopulmonary efficiency, gait speed, static and dynamic balance), and fall risk reduction. Early on, exercise was not recommended for persons diagnosed with MS as strenuous physical activity was perceived as a potential risk to accelerate disease progression (Li et al., 2022). More recent data such as a Mendelian randomization study indicate, however, that moderate physical activity correlates with a lower risk for MS (Li et al., 2022).

A notable drawback of many exercise-centered studies conducted in pwMS is that they are mostly based on small trials with few patients and are thus consistently underpowered. Nonetheless, the extensive research on exercise physiology over the last few decades has sparked interest in exercise interventions as adjunct therapy (Dalla Costa and Comi, 2022; Faraclas, 2023; Gitman et al., 2023; Reina-Gutiérrez et al., 2023). It is well accepted that exercise can increase the diameter of muscle fibers, boost mitochondrial function, and enhance phosphocreatine recovery in healthy individuals and people with a range of health conditions. In pwMS, exercise increases myofiber size (Dalgas et al., 2010; Wens et al., 2015) and enhances phosphocreatine recovery (Orban et al., 2019). More importantly, however, a growing body of evidence suggests that exercise provides functional benefits by improving muscle strength, fatigue resistance, balance, mobility, cardiorespiratory fitness, and quality of life in pwMS with EDSS ≤6.5 (Hoang et al., 2022) (Table 1; Supplementary Table S1). Evidence for exercise effects at higher disability levels is scarcer and less conclusive, but a systematic review on patients with an EDSS >6 concluded that in 9 of the 13 studies reviewed, exercise improved disability, physical fitness, physical function, and/or symptomatic and participatory outcomes (Edwards and Pilutti, 2017). These findings suggest that the right form and dose of exercise can have beneficial effects on the physical and emotional health and wellbeing of pwMS.

**TABLE 1:** Summary of individual studies on different exercise modalities in pwMS.

N CG/EG	EDSS Average	Type of MS	Type of exercise (RT or AT)	Duration (weeks)	Improved functional parameter (s)	Reference
12/18	3.2	RRMS and SPMS	RT	10	Strength in lower limb (MVIC) Hand grip strength Gait speed (10-MWT (s)) Walking distance (6-MTW (m)) Fatigue resistance	Andreu-Caravaca et al. (2022a)
12/18	3.2	RRMS and PPMS	RT	10	STS TUG	Andreu-Caravaca et al. (2022b)
13/14	<4	RRMS	Combined AE + RT	12	Strength in lower limb (MVIC) 1-RM leg extension	Correale et al. (2021)
13/14	1.5	RRMS	RT	12	Hip strength (MVIC) Walking distance (6-MTW (m)) Walking speed (T25FW (s)) Fatigue resistance	Keller et al. (2021)
13/13	2.38	RRMS	RT	6	Strength in lower limb (MVIC and isokinetic) TUG Fatigue resistance	Gomez-Illan et al. (2020)
20/23	3.5–4	Not specified	RT	10	Strength in lower limb Walking distance (6-MTW (m)) Fatigue resistance	Callesen et al. (2020)
15/19	2.35	RRMS	RT	8	Strength in lower limb (MVIC) Mobility	Moghadasi et al. (2020)

AE, aerobic exercise; RT, resistance exercise; Combined AE and RT, aerobic exercise followed by a resistance training in every training session; CG, control group; EG, experimental group; MVIC, maximal voluntary isometric contraction; MWT, minute walk test; STS, sit-to-stand; TUG, timed up and go; T25FW, timed 25-foot walk.

The exact nature of the beneficial effect of exercise depends on the type, volume, intensity and duration of the exercise regimen. Aerobic endurance exercise for 12 months increased VO_2_ peak, decreased fatigue and improved gait parameters in patients with RRMS (Schmidt and Wonneberger, 2014; Wonneberger and Schmidt, 2015). In a recent review covering 14 studies with aerobic exercise, 7 studies reported increased VO_2_, and 9 studies reported changes in perceived fatigue post-training (Taul-Madsen et al., 2021) (Supplementary Table S1).

Of particular therapeutic interest is the fact that resistance training modalities can be safe and have the potential to counter muscle weakness in pwMS. We have therefore retrieved topical summaries about resistance exercise in MS from PubMed using “resistance training” and “multiple sclerosis” as search criteria in combination with the search filters “review” and “meta-analysis.” A selection of the latest and most comprehensive summaries is provided in Supplementary Table S1 (Taul-Madsen et al., 2021; Andreu-Caravaca et al., 2023; Cano-Sánchez et al., 2024). The significance of some individual resistance exercise datasets remains hard to judge given the small number of participants. Despite this limitation, increased muscle strength was repeatedly demonstrated in pwMS following resistance training. More recent studies, i.e., from 2020 onwards, are summarized in Table 1. They were identified through a PubMed database search using the terms “multiple sclerosis,” “exercise,” and “muscle strength.” Studies that employed resistance training interventions with a minimum of 10 patients per group were exclusively selected. These latter studies corroborate that there is a consistent, significant benefit of resistance training on muscle strength improvement in pwMS. Apart from one (Andreu-Caravaca et al., 2022b), all studies reported increased maximum voluntary isometric contractions (MVIC) of the lower limb muscles (Callesen et al., 2020; Gomez-Illan et al., 2020; Moghadasi et al., 2020; Correale et al., 2021; Keller et al., 2021; Andreu-Caravaca et al., 2022a). MVIC is a standard method to measure muscle strength and is predictive of mobility-related parameters (Valenzuela et al., 2020). Resistance training is associated with improvements in the Timed-Up-and-Go (TUG) and 6-min walk test (6-MWT) (Callesen et al., 2020; Gomez-Illan et al., 2020; Keller et al., 2021; Andreu-Caravaca et al., 2022a; Andreu-Caravaca et al., 2022b; Andreu-Caravaca et al., 2023). Several studies report additional, beneficial effects of resistance exercise on general fatigue (Akbar et al., 2020; Callesen et al., 2020; Gomez-Illan et al., 2020; Englund et al., 2022).

## The beneficial effects of exercise as an indicator of potential drug targets in MS

While exercise can improve musculoskeletal health in pwMS, it is important to mention that not every patient can engage in moderate to strenuous exercise or physical activity and that the long-term adherence to exercise interventions is difficult to maintain. This is particularly relevant for pwMS where MS-associated muscle weakness, increased muscle fatigue, and general fatigue can result in a reduction in one’s activity level (Oliva Ramirez et al., 2021; Zheng et al., 2023). It has been hypothesized that pwMS engage in less physical activity than healthy individuals resulting in disuse and deconditioning of skeletal muscle over time (Sandroff et al., 2015; Macdonald et al., 2023). Drugs that mimic the plastic adaptations to physical activity could thus constitute another treatment option. However, the high complexity of exercise has hampered the unraveling and pinpointing of a primary pathway that mimics the holistic effects of exercise (Sanford et al., 2020; Furrer et al., 2023). Notwithstanding, targeting critical mediators of physical activity could constitute a promising approach to support, facilitate and/or amplify key adaptations that occur in response to regular exercise. Currently, this area of research is actively being pursued preclinically and clinically (Sanford et al., 2020).

In response to exercise, several hundreds of substances, including bioactive proteins or metabolites are secreted. These substances, collectively referred to as exerkines (or myokines if they originate from skeletal muscle), can act in an autocrine, paracrine, or endocrine manner to coordinate the local and systemic processes that drive adaptations to exercise across various tissues (Furrer et al., 2023; Pedersen, 2023). Accordingly, they have potential to not only improve musculoskeletal capability and capacity, but also neurological and other functions that are impaired in pwMS.

## Discussion

Based on the current knowledge, appropriate exercise can be a safe and beneficial treatment for pwMS, provided individuals have no comorbidities that prevent them from participating in exercise interventions (Learmonth et al., 2023). Most exercise data have been acquired from patients that were still mobile and able to walk at least shorter distances. One can derive from these data that exercise interventions should ideally be implemented as early as possible to help patients maintain and possibly improve their level of mobility and muscle function. This thinking is corroborated by comprehensive investigations into how patients acquire disability. The analysis expounds pre-existing disability and older age as principal risk factors for further disability accumulation (Lublin et al., 2022). Thus, early intervention is best placed to maintain or improve current levels of physical function and delay disability accrual by years. Aerobic exercise can be prescribed to patients suffering from generalized fatigue (Figure 1) and has beneficial effects on walking capacity and cardiorespiratory fitness with few intramuscular metabolic aspects (e.g., phosphocreatine recovery) and a limited effect on improving muscle strength in pwMS. By contrast, there is compelling evidence that resistance training efficiently counters muscle weakness and restores parameters associated with mobility in patients with mild to moderate disability (Figure 1). Moreover, resistance exercise can improve the perception of general fatigue. Thus, resistance exercise targets two debilitating factors in pwMS, muscle weakness and fatigue. Therefore, the data suggest the health effects of both aerobic and resistance training exercise are safe and offer complementary benefits for pwMS.

Many effects of exercise, and particularly of resistance exercise, are mediated via the release of bioactive exerkines from skeletal muscle (e.g., myokines or myometabokines). These secreted substances can act in an autocrine, paracrine, or endocrine manner on multiple tissues (Rai and Demontis, 2022). These substances have the potential to confer broader health benefits by targeting neuronal and musculoskeletal dysfunctions in pwMS at the same time. Modulation of exerkines and/or their downstream effectors could open a new therapeutic avenue to mimic specific aspects of exercise and improve multiple symptoms associated with MS (i.e., mobility disability).
