# Supplementary material for: The therapeutic potential of exercise for improving mobility in multiple sclerosis
Source: Front Physiol. 2024 Sep 13;15:1477431. doi: 10.3389/fphys.2024.1477431 (PMC11427913; doi:10.3389/fphys.2024.1477431)
Supplement: Supplementary file 1 [file Table1.pdf]

Supplemental Table 1: Reviews and meta-analysis of exercise studies assessing musculoskeletal parameters in pwMS

Type of Exercise (RT or AT)	Duration (weeks)	Improved Functional Parameter(s)	Number of Studies with Improvements/Total Number of Studies	Reference
AE	3-26	VO_2 peak, walking capacity (6MWT)	7/14	(Taul-Madsen et al., 2021)
RT	8-24	Muscle strength	7/7	(Taul-Madsen et al., 2021)
RT	5-24	Muscle strength<br>Gait	5/6<br>4/8	(Cano-Sánchez et al., 2024)
RT	> 6	Strength in lower limb<br>Gait speed<br>Walking distance<br>TUG<br>Fatigue resistance	n/a<br>(meta-analysis)	(Andreu-Caravaca et al., 2023)

AE: Aerobic Exercise, RT: Resistance Exercise, TUG: Timed Up and Go
